# Automated abdominal aortic calcification scoring from vertebral fracture assessment images and fall-associated hospitalisations: the Manitoba Bone Mineral Density Registry

**DOI:** 10.1007/s11357-025-01589-7

**Published:** 2025-03-13

**Authors:** Marc Sim, Abadi K. Gebre, Jack Dalla Via, Siobhan Reid, Mohammad Jafari Jozani, Douglas Kimelman, Barret A. Monchka, Syed Zulqarnain Gilani, Zaid Ilyas, Cassandra Smith, David Suter, John T. Schousboe, Joshua R. Lewis, William D. Leslie

**Affiliations:** 1 School of Medical and Health Sciences, Nutrition & Health Innovation Research Institute, Edith Cowan University, Perth, WA 6027 Australia; 2 Medical School, The University of Western Australia, Perth, Australia; 3 Department of Computer Science, Concordia University, Montreal, Canada; 4 Department of Statistics, University of Manitoba, Winnipeg, Canada; 5 Department of Radiology, Rady Faculty of Health Sciences, University of Manitoba, Winnipeg, Canada; 6 George and Fay Yee Centre for Healthcare Innovation, University of Manitoba, Winnipeg, Canada; 7 Centre for AI&ML, School of Science, Edith Cowan University, Perth, Australia; 8 Department of Computer Science and Software Engineering, The University of Western Australia, Perth, Australia; 9 Park Nicollet Clinic and HealthPartners Institute, HealthPartners, Minneapolis USA; 10 Division of Health Policy and Management, University of Minnesota, Minneapolis, USA; 11 Departments of Medicine and Radiology, University of Manitoba, Winnipeg, Canada

**Keywords:** Vascular calcification, Subclinical cardiovascular disease, Injurious falls, Vertebral fracture assessment, Machine learning

## Abstract

Abdominal aortic calcification (AAC), a subclinical measure of cardiovascular disease (CVD) that can be assessed on vertebral fracture assessment (VFA) images during osteoporosis screening, is reported to be a falls risk factor. A limitation to incorporating AAC clinically is that its scoring requires trained experts and is time-consuming. We examined if our machine learning (ML) algorithm for AAC (ML-AAC24) is associated with a higher fall-associated hospitalisation risk in the Manitoba Bone Mineral Density (BMD) Registry. A total of 8565 individuals (94.0% female, age 75.7 ± 6.8 years) who had a BMD and VFA image from DXA between February 2010 and December 2017 were included. ML-AAC24 was categorised based on established categories (ML-AAC24 = low < 2; moderate 2 to < 6; high ≥ 6). Cox proportional hazards models assessed the relationship between ML-AAC24 categories and incident fall-associated hospitalisations obtained from linked health records (mean ± SD follow-up, 3.9 ± 2.2 years). Individuals with moderate (9.6%) and high ML-AAC24 (11.7%) had a greater proportion of fall-associated hospitalisations, compared to those with low ML-AAC24 (6.0%). In age and sex-adjusted models, compared to low ML-AAC24, moderate (HR 1.49, 95% CI 1.24–1.79) and high ML-AAC24 (HR 1.89, 95% CI 1.56–2.28) were associated with greater hazards for a fall-associated hospitalisation. Results were comparable (HR 1.37, 95% CI 1.13–1.65 and HR 1.60, 95% CI 1.31–1.95, respectively) after multivariable adjustment, including prior falls and CVD, as well as medication use. Integrating ML-AAC24 into bone density machine software to identify high risk individuals would opportunistically provide important information on fall and cardiovascular disease risk to clinicians for evaluation and intervention.

**Supplementary Information:**

The online version contains supplementary material available at 10.1007/s11357-025-01589-7.

## Introduction

Among individuals aged over 70 years, falls are the predominant cause of injury-related hospitalisations and fatalities worldwide [1]. Approximately 10–20% of fallers experience injuries such as fractures or traumatic brain injuries that require urgent medical attention [2]. In conjunction with other consequences such as pain, loss of mobility, and independence [3], this likely contributes to the high hospitalisation costs of falls. For example, in the USA, the average cost of a fall hospitalisation in 2015 was $30,550 [4], while another study reported an average cost of $62,521 in 2019 [5]. As falls are a major cause of osteoporotic fractures, this contributes heavily to the 4.3 million fragility fractures that cost the healthcare systems of Europe over €56 billion annually based on data from 2019 [6]. This highlights the need to comprehensively identify novel fall risk factors [7] to enable early intervention (e.g. clinical assessment, exercise, nutrition).

Despite being often overlooked, there is emerging evidence linking asymptomatic and clinical cardiovascular disease (CVD) as risk factors for falls and associated injuries [8–10]. Abdominal aortic calcification (AAC) is one measure of subclinical CVD that can be identified using lateral spine images (LSI) during routine osteoporosis screening as part of vertebral fracture assessment (VFA) [11, 12]. Obtaining these low-cost dual-energy X-ray absorptiometry (DXA)–derived VFA images allows AAC assessment for early stratification of individuals at high risk of CVD [12]. Noteworthy, AAC can also be assessed from lateral spine radiographs (X-ray) and quantitative computed tomography (CT) [13]. Although lateral spine radiographs are considered the “gold standard” for VFA, DXA-derived LSI are often used to detect vertebral fractures with a reported sensitivity of 70–93% and specificity of 95–100% [14]. The widespread clinical use of DXA for osteoporosis screening and its low radiation dose make it an ideal screening tool. This would be especially relevant to older women who are most likely to undertake bone density assessment due to their high risk for osteoporosis and falls [15].

Typically scored on a well-established 24-point semi-quantitative scale (AAC24) [13], AAC strongly predicts CVD risk [16]. Previously, it was reported that the presence (and/or severity) of AAC is linked with a greater 5-year decline in muscle strength [17], lower bone mineral density (BMD), and fracture risk [18]. It was also reported that AAC was associated with increased long-term injurious fall risk in community-dwelling older women [19]. Such findings are unlikely to be a result of a single mechanism, but perhaps the collective impact of compromised vascular health on multiple organ systems (e.g. musculoskeletal, neurological). Despite the potential use of AAC as a marker of musculoskeletal and cardiovascular health, a major limitation to integrating AAC assessment into routine clinical practice is that it requires highly trained readers to manually score it from VFA images. This is a time-consuming process, taking experts between 5 and 6 min per image [13]. Unsurprisingly, emerging technological advances such as the use of quantitative computer-assisted tracking of calcifications (QC) on radiographs of the lateral spine [20], as well as convolutional neural networks applied to DXA-derived LSI [21], have been shown to reduce the time/resources taken for accurate AAC assessment. For example, QC software application enables interactive visualisation and tracing of the calcified tracts on radiographs [20]. The use of technology is an important step to enhancing the clinical utility of such images.

Using machine learning, our team recently developed an algorithm that can detect and instantly provide automated AAC24 scores (ML-AAC24) from VFA obtained from four of the most commonly used DXA machines globally (GE Lunar Prodigy and iDXA; Hologic 4500A and Horizon) [21]. The validity of our ML-AAC24 algorithm, categorised according to severity (low, moderate, high), has also been reported when considering the risk of cardiovascular events and mortality [21]. Using this ML-AAC24 on VFA images captured on a Hologic 4500A bone density machine, we recently reported that moderate to high ML-AAC24, compared to low ML-AAC24, is associated with 35% greater long-term fall-related hospitalisation risk in 1023 community-dwelling older women (mean age 75 ± 3 years) [22], replicating our earlier findings when AAC was scored manually [19].

Despite these novel results, a limitation of the aforementioned work [19] is that ML-AAC24 was developed on the same set of images examined for its relationship with falls. Therefore, it is essential to replicate these findings as part of a real-world clinical setting, where VFA images are routinely captured in high risk populations such as older women [22]. To date, no study using VFA images derived from GE manufactured DXA machines has investigated if AAC is associated with increased fall risk. The current study examined the relationship between ML-AAC24 and fall-associated hospitalisations within the Manitoba Bone Mineral Density Registry, representing a real-world clinical setting that uses DXA machines manufactured by GE (Prodigy and iDXA).

## Materials and methods

### Manitoba registry

The Manitoba Bone Mineral Density Program manages all clinical DXA testing in the Province of Manitoba, Canada, and maintains a registry of all results. The Program includes VFA images in DXA assessment since 2010 for qualifying individuals using the criteria T-score of ≤ − 1.5 (minimum at the lumbar spine, total hip, or femoral neck) plus any of (a) age ≥ 70 years, (b) age 50 to 69 years and historical height loss (recalled young adult height minus current height) > 5 cm or measured height loss > 2.5 cm, or (c) glucocorticoid exposure for at least 3 months over the past year. All scans were performed using fan-beam DXA instruments (Lunar Prodigy or iDXA, GE Healthcare, Madison, WI). From the initial 13,395 de-identified VFA images obtained as part of osteoporosis assessment from February 2010 to December 2017, 4830 were excluded for various reasons (e.g. no linked health records, poor image quality, images from the same patient), as detailed in Supplementary Fig. 1. This left 8565 unique individuals with a VFA image where the ML-AAC24 algorithm was applied for inclusion into the current study.

### AAC assessment and development

De-identified VFA images were available from two different models (Lunar Prodigy, iDXA) of bone density machines manufactured by GE Healthcare (Madison, WI). The ML-AAC24 scores were developed based on the Kaupilla AAC24 [23] semi-quantitative scoring method undertaken by an expert and described in detail previously [21]. To manually score AAC24, the posterior and anterior walls of the abdominal aorta are divided into four segments each, corresponding to the spaces anterior to vertebral levels L1, L2, L3, and L4. Within these eight segments, the AAC score was assigned as follows: 0 if no calcification was observed, 1 if less than one third of the aortic wall length in that segment was calcified, 2 if one third or more but less than two-thirds of the aortic wall length was calcified, and 3 if two-thirds or more of the aortic wall length was calcified. The total AAC score, ranging from 0 to 24, is calculated as the sum of the scores from the eight segments. The AAC24 scoring method is the most used method with higher intra-rater reliability (intraclass correlations coefficients (ICC) above 0.9) compared to AAC-8 scoring (ICC 0.8–0.9) [23, 24]. Expert scores served as the ground truth for the ML-AAC24 algorithm training (*n* = 2590) [21]. Specifically, the ML-AAC24 algorithm was developed on a randomly chosen subset comprising 90% of images (*n* = 2331) of those with expert AAC readings, and subsequently, it was tested on an additional 10% of images (*n* = 259). This process was iterated through tenfold cross-validation to predict AAC scores for each 10% random sample (*n* = 259). The intraclass correlations between expert and ML-AAC24 readings were 0.90 and 0.79 for VFA images acquired using iDXA and Lunar Prodigy DXA machines, respectively. Similar to previous reports on AAC classification based on severity [12, 21], ML-AAC24 was categorised as low (ML-AAC24 < 2), moderate (ML-AAC24 ≥ 2 to < 6), and high (ML-AAC24 ≥ 6). The performance of ML-AAC24 to classify individuals into the three ML-AAC24 severity groups was detailed previously [21]. Specifically, average classification accuracy (76.6%), sensitivity (60.4%), specificity (81.9%), negative (81.9%), and positive (63.0%) predictive values were acceptable for the Lunar Prodigy. For the iDXA, average classification accuracy (85.0%), sensitivity (74.2%), specificity (88.8%), negative (88.9%), and positive (74.0%) predictive value were better.

The final algorithm, which is an ensemble of ten convolutional neural networks derived from the aforementioned subsets, was then applied to estimate ML-AAC24 for 8565 VFAs (without expert readings or used in the development of the algorithm) for inclusion in the current study.

### Fall-associated hospitalisation

The primary outcome for this study was fall-associated hospitalisation after VFA (index date) up until the 31st of March 2018. We also identified individuals with a prior fall using a look back of fall-associated hospitalisation diagnoses or self-reported falls that occurred in the 12 months prior to the index date. Data for incident fall-associated hospitalisations were based upon hospital discharge abstract codes ((ICD-9-CM) prior to 2004 and International Classification of Diseases, 10th Revision, Canadian Enhancements (ICD-10-CA)). Fall codes included E880–886, 888 for ICD-9-CM, and W00-W19 for ICD-10-CA. Incident fall-associated hospitalisations were assessed from routine prospectively collected linked administrative data, independent of self-report.

### Baseline characteristics

Multiple covariates that could contribute to CVD and musculoskeletal health were identified and included in the adjusted models. This included age, sex, body mass index (BMI), current tobacco smoking, high alcohol intake (3 or more drinks per day) assessed at the time of VFA, diagnoses of diabetes, chronic kidney disease (CKD) hypertension in the last 3 years (linked physician claims and hospitalisation records), and prior myocardial infarction or cerebrovascular disease at any time since 1984 (linked hospitalisation records). Social determinants related to income (lower two quintiles versus upper three quintiles), area of residence (rural versus urban), and ethnicity (self-reported white versus non-white) were also included [25, 26]. Medications used for at least 6 months in the year prior to the index date were identified from the province-wide retail pharmacy database (use of any spironolactone, glucocorticoid, statin, nonselective beta blocker, selective beta blocker, angiotensin receptor blocker, ACE inhibitor, aldosterone blocker, loop diuretic, thiazide diuretic, digoxin, calcium channel blocker, long-acting nitrate, diabetes medications, oral anticoagulant) [27, 28].

### Statistical analysis

Kaplan–Meier survival analysis was used to determine the relationship of ML-AAC24 categories (low, moderate, and high) with incident fall-associated hospitalisations. This relationship was further examined using multivariable-adjusted Cox proportional hazards models. Three models of adjustment were included: Model 1 adjusted for age and sex; Model 2 adjusted for Model 1 plus BMI, tobacco use, high alcohol intake, income, rural residence, ethnicity, diagnoses of diabetes, hypertension, and medication use in the prior year (glucocorticoid, statin, nonselective beta blocker, selective beta blocker, angiotensin receptor blocker, ACE inhibitor, aldosterone blocker, loop diuretic, thiazide diuretic, digoxin, calcium channel blocker, long-acting nitrate, and oral anticoagulant); and Model 3: Model 2 plus prior falls in the last year. The proportional hazards assumption was confirmed from graphical analyses and Schoenfeld residuals. The log-likelihood chi-square statistic was used to evaluate the significance of removing individual predictor variables from the multivariable-adjusted analysis (Model 3). The resulting change in log-likelihood chi-square for each predictor variable was used for ranking its relative importance in relation to falls. A larger change in log-likelihood chi-square upon the removal of a variable from the final model indicates a variable of greater importance. All analyses were performed using IBM SPSS (Version 27; IBM Corp., Armonk, NY, USA)*.*

### Sensitivity analyses

As lower BMD [29] and CVD [9] have been linked to increased fall risk, we further adjusted the primary analysis for hip BMD as well as prior major CVD, comprising myocardial infarction (ICD-9-CM 410, ICD-10-CA I21) or cerebrovascular disease (ICD-9-CM 433–435, ICD-10-CA I63-66, G45) from linked health records since 1984. As diabetes medications and CKD can influence vascular calcification [16], these variables were also added as covariates to Model 3 when examining the relationship between ML-AAC24 categories and fall-associated hospitalisations. As individuals with prior falls likely have a much higher fall risk, which could bias our results, further analysis excluding these prior fallers (*n* = 1332) as part of the primary analysis between ML-AAC24 categories and fall-associated hospitalisations was undertaken. Although the proportion of males in this study was low (6.0%), we undertook exploratory sex-specific analysis, including interaction testing (sex*ML-AAC24 categories), to determine if the relationship between ML-AAC24 and fall-associated hospitalisations was influenced by sex.

## Results

Baseline cohort characteristics presented by ML-AAC24 severity categories are displayed in Table 1. Mean ± SD age of the cohort was 75.7 ± 6.8 years, with 94.0% of all individuals being female. A total of 3400 (39.7%), 2840 (33.2%), and 2325 (27.1%) individuals presented with low, moderate, and high ML-AAC24, respectively. Individuals with high ML-AAC24 were older compared to those with low and moderate ML-AAC24 (by 4.9 and 2.0 years, respectively). BMI appeared comparable across ML-AAC24 categories. A greater proportion of individuals had diabetes, hypertension, and prior falls with increasing severity of ML-AAC24. A similar trend was also observed when considering medications, with those with high ML-AAC24 generally recording higher usage across most medications (except glucocorticoids).

**Table 1:** Baseline characteristics by abdominal aortic calcification score categories obtained using machine learning (ML-AAC24)

	All participants (*n* = 8565)	Predicted AAC categories (ML-AAC24)^a^			
		Low (*n* = 3400, 39.7%)	Moderate (*n* = 2840, 33.2%)	High (*n* = 2325, 27.1%)	
Demographics					
Age, years	75.7 ± 6.8	73.4 ± 6.4	76.3 ± 6.5		78.3 ± 6.5
Sex, female	8052 (94.0)	3242 (95.4)	2642 (93.0)		2168 (93.2)
Non-white ethnicity	353 (4.1)	173 (5.1)	111 (3.9)		69 (3.0)
Body mass index (BMI), kg/m^2^	26.2 ± 5.0	26.2 ± 5.1	26.6 ± 5.0		25.8 ± 4.7
Diabetes^b^	1111 (13.0)	316 (9.3)	390 (13.7)		405 (17.4)
Hypertension^b^	4678 (54.6)	1497 (44.0)	1626 (57.3)		1555 (66.9)
Tobacco use	691 (8.1)	134 (3.9)	227 (8.0)		330 (14.2)
High alcohol intake	19 (0.2)	S	S		S
Lower income	2791 (32.6)	997 (29.3)	915 (32.2)		879 (37.8)
Rural residency	2265 (26.4)	824 (24.2)	788 (27.8)		653 (28.1)
Falls in the year prior to index date	1332 (15.6)	439 (12.9)	463 (16.3)		430 (18.5)
Medication use in the year prior to index date					
Glucocorticoid	502 (8.9)	187 (5.5)	181 (6.4)		134 (5.8)
Statin	2667 (31.1)	768 (22.6)	907 (31.9)		995 (42.8)
Nonselective beta blocker	148 (1.7)	40 (1.2)	54 (1.9)		54 (2.3)
Selective beta blocker	1249 (14.6)	308 (9.1)	412 (14.5)		529 (22.8)
Angiotensin receptor blocker	1497 (17.5)	460 (13.5)	534 (18.8)		503 (21.6)
ACE inhibitor	1490 (17.4)	444 (13.1)	526 (18.5)		520 (22.4)
Aldosterone blocker	86 (1.0)	19 (0.6)	21 (0.7)		46 (2.0)
Loop diuretic	477 (5.6)	105 (3.1)	173 (6.1)		199 (8.6)
Thiazide diuretic	1283 (15.0)	418 (12.2)	441 (15.5)		424 (18.2)
Digoxin	103 (1.2)	24 (0.7)	35 (1.2)		44 (1.9)
Calcium channel blocker	1666 (19.5)	467 (13.7)	559 (19.7)		640 (27.5)
Long-acting nitrate	128 (1.5)	18 (0.5)	45 (1.6)		65 (2.8)
Oral anti-coagulants	324 (3.8)	83 (2.4)	119 (3.9)		122 (5.2)

Data expressed as mean ± SD or *n* (%)

*S*, suppressed small cell count

^a^ML-AAC-24 groups, low < 2, moderate 2 to < 6, high ≥ 6

^b^Based on diagnosis codes in the last 3 years

### Fall-associated hospitalisation

**Fig. 1:**
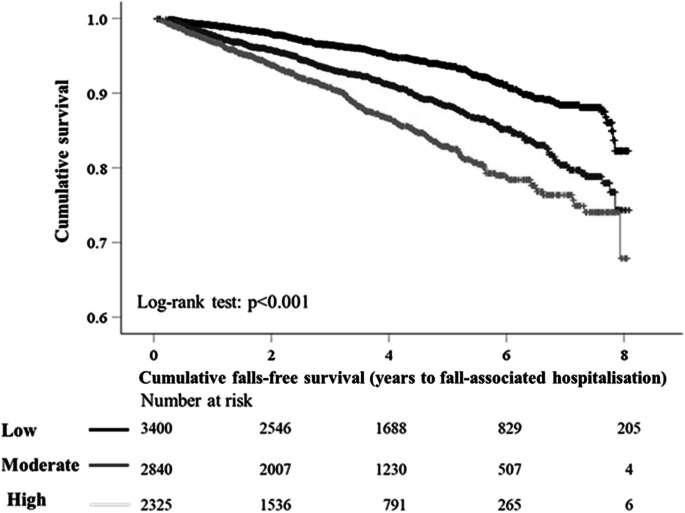
Kaplan-Maier survival curve for the relationship between predicted abdominal aortic calcification scores (ML-AAC24) by severity and fall-associated hospitalisations. Black, dark grey, and light grey lines represent low, moderate, and high ML-AAC24, respectively

**Fig. 2:**
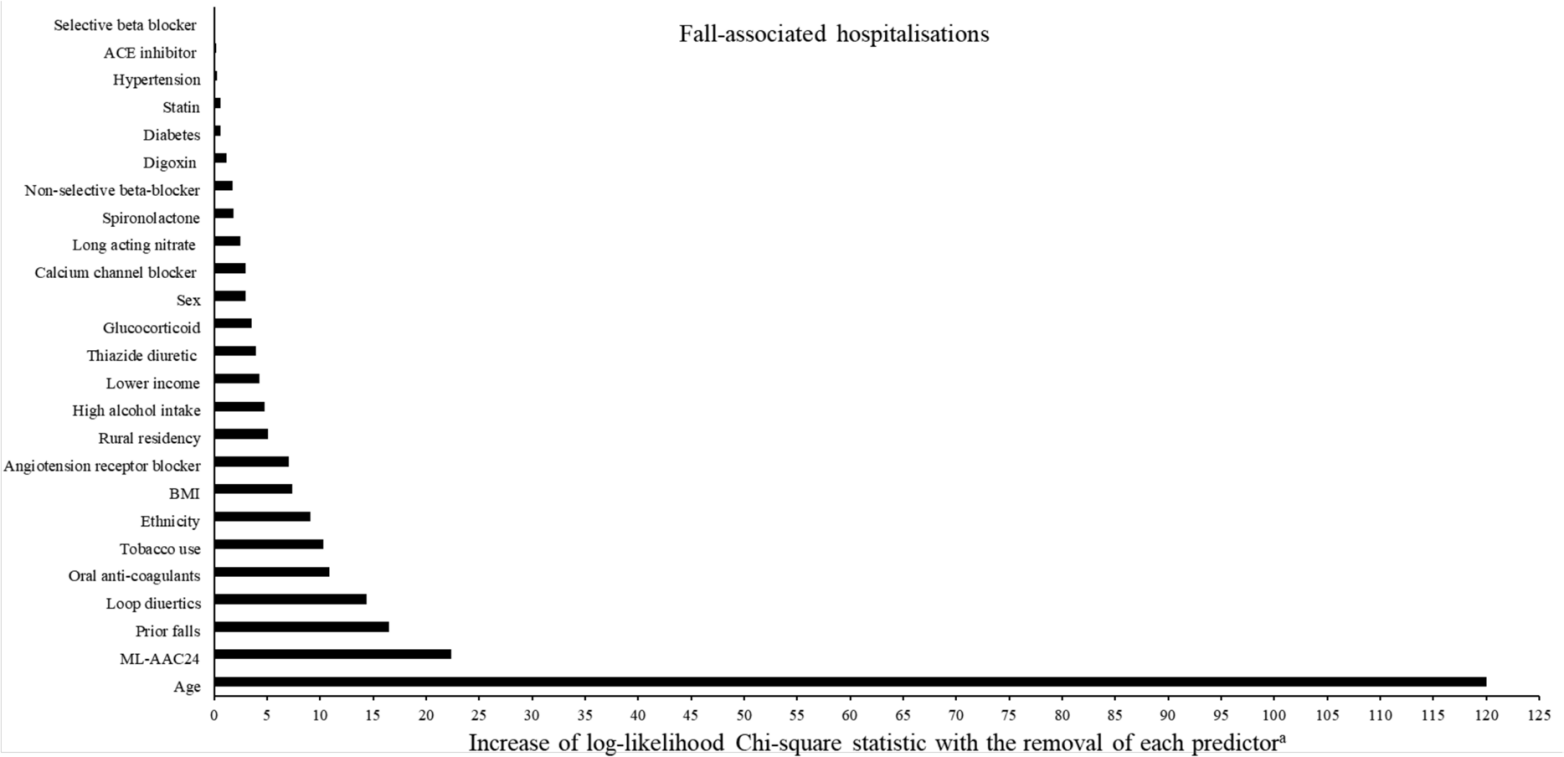
Ranking order for the importance of variables in the multivariable-adjusted analysis (Model 3) when considering the risk for a fall-associated hospitalisation. ^a^Larger change in log-likelihood chi-square statistic (*χ*^2^) after the removal of the predictor from the final model indicates greater importance to the model

Over a maximum of 8 years (33,403 person years) of follow-up (mean ± SD, 3.9 ± 2.2 years), 8.8% (750/8565) of participants experienced a fall-associated hospitalisation. Compared to low ML-AAC24, the proportion of fall-associated hospitalisations recorded was almost double in high ML-AAC24 (6.0% vs. 11.7%, respectively). Kaplan-Maier survival curves for the relationship between ML-AAC24 categories and fall-associated hospitalisations are presented in Fig. 1. When adjusted for age and sex (Model 1), moderate (HR 1.49, 95% CI 1.24–1.79) and high (HR 1.89, 95% CI 1.56–2.28) ML-AAC24 recorded greater hazards for a fall-associated hospitalisation, compared to low ML-AAC24 (Table 2). After adjustment for a range of risk factors related to lifestyle and medication use (Model 2), the hazard ratios comparing moderate (HR 1.39, 95% CI 1.15–1.67) and high (HR 1.63, 95% CI 1.34–1.99) ML-AAC24 to low ML-AAC24 remained statistically significant but were slightly lower. Further adjustment for prior falls did not alter point estimates (Model 3). Based on the Wald *χ*^2^ statistic, the relative importance of all predictors (as part of Model 3) when considering the relationship between ML-AAC24 and fall-associated hospitalisations is presented in Fig. 2. The largest change in log-likelihood chi-square (*χ*^2^) with the removal of the variable from Model 3 was recorded for age (*χ*^2^ = 120.0), ML-AAC24 (*χ*^2^ = 22.4), prior falls (*χ*^2^ = 16.5), loop diuretic use (*χ*^2^ = 14.4), and anticoagulant use (*χ*^2^ = 10.9), indicating they were the top five predictors.

**Table 2:** Hazard ratios (95% CI) for fall-associated hospitalisation by machine learning–derived abdominal aortic calcification 24 scores (ML-AAC-24) categorised by severity

Predicted ML-AAC-24	Fall-associated hospitalisations, *n* (%)	Model 1	Model 2	Model 3
Low, *n* = 3400	205 (6.0)	Ref (1.0)	Ref (1.0)	Ref (1.0)
Moderate, *n* = 2840	272 (9.6)	1.49 (1.24–1.79)*	1.39 (1.15–1.67)*	1.37 (1.13–1.65)*
High, *n* = 2325	273 (11.7)	1.89 (1.56–2.28)*	1.63 (1.34–1.99)*	1.60 (1.31–1.95)*

Model 1 adjusted for age and sex; Model 2 adjusted for Model 1 + body mass index, tobacco use, high alcohol intake, income, rural residence, ethnicity, diagnoses of diabetes, hypertension, medication use in the year prior to index date (glucocorticoid, statin, nonselective beta blocker, selective beta blocker, angiotensin receptor blocker, ACE inhibitor, aldosterone blocker, loop diuretic, thiazide diuretic, digoxin, calcium channel blocker, long-acting nitrate, and oral anticoagulant); and Model 3: Model 2 + falls in the year prior to index date

^*^p < 0.05 compared to low ML-AAC24

### Sensitivity analyses

Additional adjustment for hip BMD and prior myocardial infraction or cerebrovascular events to Model 3 did not alter the observed relationship between ML-AAC24 categories and fall-associated hospitalisation (Supplementary Table 1). Specifically, those with moderate (HR 1.33, 95% CI 1.10–1.60) and high (HR 1.52, 95% CI 1.25–1.86) ML-AAC24 had greater hazards for a fall-associated hospitalisation compared to low ML-AAC24. The addition of diabetes medications (HR 1.06, 95% CI 0.72–1.56) and CKD (HR 1.60, 95% CI 1.19–2.16) did not influence the relationship between ML-AAC24 and fall-associated hospitalisations. Specifically, compared to low ML-AAC24, those with moderate (HR 1.37, 95% CI 1.13–1.65) and high ML-AAC24 (HR 1.58, 95% CI 1.29–1.93) still had greater hazards for a fall-associated hospitalisation. After excluding individuals with a prior fall (*n* = 1332), those with moderate (HR 1.45, 95% CI 1.18–1.78) and high ML-AAC24 (HR 1.63, 95% CI 1.31–2.03) continued to record greater hazards for a fall-associated hospitalisation (Model 3). No interaction for sex*ML-AAC24 category was observed in all three models of adjustment (*p* > 0.1) when considering the relationship between ML-AAC24 categories and fall-associated hospitalisations. When stratified by sex, no relationship was observed between ML-AAC24 categories and fall-associated hospitalisations in men (*n* = 512) (Supplementary Table 2). For women (*n* = 8053), compared to low ML-AAC24, those with moderate and high ML-AAC24 had greater hazards for a fall-associated hospitalisation.

## Discussion

In a predominantly older female cohort, we demonstrate that over 1 in 2 individuals attending osteoporosis screening had moderate or high ML-AAC24. These individuals also had higher fall-associated hospitalisation risk (37% and 60%, respectively) compared to those with low ML-AAC24. Notably, the observed relationships were independent of numerous fall risk factors, including medication use and prior fall history. Results remained materially similar after further adjustment for prior myocardial infarction or cerebrovascular disease, as well as hip BMD. It was noteworthy that ML-AAC24 was only behind age when considering the relative predictive importance of all variables within the multivariable-adjusted analysis. These results highlight the potential clinical utility of ML-AAC24 to identify individuals with higher fall risk for early referral to appropriate fall prevention interventions.

We have previously reported that AAC assessed by human experts and automated algorithms [19, 22] is related to greater fall-related hospitalisation risk in older women (*n* = 1023, mean age ± SD 75 ± 3 years) from the general population. While the present study is based on a clinical registry involving a relatively large population of older women and men that underwent VFA as part of routine clinical care, the observed hazard ratios (HRs) are comparable with our previous study in women only. In our previous work, women with moderate to high ML-AAC24 had a multivariable-adjusted HR of 1.30 (95% CI 1.04–1.61) and 1.72 (95% CI 1.19–2.47), respectively [22]. In comparison, the multivariable-adjusted hazards in the current study were similar for the moderate (HR 1.37, 95% CI 1.13–1.65) and high ML-AAC24 (HR 1.60, 95% CI 1.31–1.95). Of note, in exploratory sex-specific analysis in the current study, ML-AAC24 was only associated with fall-associated hospitalisation in women and not men. However, such findings warrant further investigation as only 6% of the current cohort were male, and no significant interaction for sex and ML-AAC24 with falls was recorded. Men also tend to present with higher AAC [30], and current cut-points for the severity of ML-AAC24 have been examined in cohorts comprising predominantly of older women [21]. As such, whether sex-specific cut-points are needed for ML-AAC24 also warrants further investigation. Nevertheless, our current findings are important as they represent external validation of ML-AAC24 as a fall risk factor within a real-world clinical registry of patients with high osteoporosis risk, a population most likely to benefit from the implementation of early fall prevention interventions. Furthermore, we have now provided evidence for ML-AAC24 identified from VFA images captured on GE manufactured bone density machines (iDXA and Lunar Prodigy). This builds on previous work demonstrating similar results when considering VFA images obtained from the Hologic 4500A [22]. Collectively, the current study and previous work demonstrate the use of ML-AAC24 as a part of routine VFA as a predictor of fall risk across both Hologic- and GE-manufactured DXA machines.

Previously, it has also been reported that other measures of subclinical CVD such as carotid atherosclerosis [8] and elevated high sensitivity cardiac troponin I [31] are related to a greater risk of falls. Therefore, current findings add to the existing evidence that subclinical CVD is an important fall risk factor. This reinforces why cardiovascular health should be considered as part of fall risk assessment [10]. Remarkably, ML-AAC24 emerged as a key contributor to fall risk just after age. It is not surprising that age was among the most important contributors to fall risk, given its well-established predictive value [32]. However, it is interesting that ML-AAC24 had a greater contribution compared to traditionally believed fall risk factors such as prior falls in the last year [33] and loop diuretics [34]. In addition to the important information regarding cardiovascular and musculoskeletal health that opportunistic AAC assessment provides, it could also extend to cognitive health, specifically as late-life dementia risk may be exacerbated by moderate and high AAC [35].

As detailed previously [18, 19], there are several plausible mechanisms by which AAC may compromise musculoskeletal health and increase fall risk. Falls are often caused by a complex interaction of multiple factors (e.g. environment, visual impairment) and pathological mechanisms, including cardiovascular-associated transient events (e.g. syncope, dizziness, hypotension) [10], generalised atherosclerosis [8, 36], weaker muscle strength [37], and cognitive impairment and dementia [38, 39]. Considering the multifactorial nature of falls, AAC is likely a marker of multisite atherosclerosis that may increase the risk of falls by exacerbating one or more of these conditions [11, 17, 35]. For example, AAC is associated with thickening and stiffening of the arterial wall (e.g. aortic stiffness) [40, 41], which in turn is associated with impairment of blood supply of essential nutrients and oxygen to vital organs, including the skeletal muscle and the brain [42, 43]. Collectively, this may impair muscle strength and balance, as well as increase the risk of cardiovascular-related transient episodes and consequently falls [44].

The findings of the present study have important clinical implications. Given that falls are a significant public health concern among older adults [45], identifying high risk individuals can lead to interventions (e.g. medication review and optimisation, exercise, diet) to prevent and manage falls. Previous reports have consistently shown that measures of subclinical CVD, including AAC [8, 19, 31], are associated with higher injurious fall risk. However, a limitation of integrating AAC assessment into clinical practice to date has been the labour-intensive nature and lack of trained readers for its assessment. Overcoming this limitation, the current study provides proof of concept that automating this process through ML-AAC24 in a real-world clinical setting provides important clinical information when considering falls.

Despite these novel results, the study has several limitations that must be acknowledged. This cohort comprises predominantly of older White women (94%), thus limiting its generalisability to other populations. Although exploratory sex-specific analysis indicates that ML-AAC24 was only associated with fall-associated hospitalisations in women, the small sample size of men (*n* = 512, 6%) and non-significant interaction for sex highlight the need for larger confirmatory studies. Nevertheless, our study had excellent population coverage, which included all Manitoba residents referred for VFA as part of osteoporosis screening [46]. Additionally, given the observational nature of the study, causality cannot be established, and the effect of unmeasured confounders cannot be excluded. However, we have considered multiple potential fall risk factors, including fall history, medication use, and prevalent diseases (e.g. hypertension, myocardial infarction, cerebrovascular disease, diabetes, CKD), to minimise the impact of confounding factors. Finally, fall-associated hospitalisation was obtained from discharge codes, which implies falls may not necessarily be the primary cause for the hospitalisation, indicating the need to cautiously interpret the findings.

The study also has important strengths. Firstly, the cohort is based on a clinical registry of patients undergoing VFA as part of osteoporosis screening for the population residing within the Manitoba region. Consequently, this represents a real-world setting which increases the generalisability of results to a clinical population where fall-associated injuries (e.g., fracture) may be exacerbated by osteoporosis. Secondly, fall-associated hospitalisations were obtained from linked health records independent of self-reports, avoiding the possibility of recall bias. Finally, we also considered detailed medication use, including several classes of cardiovascular medications and anti-coagulants, to minimise potential confounding effects. This is particularly important given cardiovascular medications may increase falls risk [34], including anti-coagulants that may increase the risk of vascular calcification and vertebral fractures [47].

## Conclusion

Moderate and high ML-AAC24 obtained from VFA images as part of routine osteoporosis screening is associated with higher fall-associated hospitalisation risk in a clinical bone density registry of predominantly older women. Given the large number of older women undergoing osteoporosis screening, integrating the automatic assessment of AAC through our recently developed machine learning algorithm (ML-AAC24) into the manufacturer’s software of commonly used bone density machines may provide clinicians with additional information to identify individuals with higher falls risk. This could enable recommendations for early targeted fall prevention interventions.

## Supplementary Information

Below is the link to the electronic supplementary material.

Supplementary file1 (DOCX 37 KB)
