# Supplementary material for: Automated abdominal aortic calcification scoring from vertebral fracture assessment images and fall-associated hospitalisations: the Manitoba Bone Mineral Density Registry
Source: GeroScience. 2025 Mar 13;47(3):4369–80. doi: 10.1007/s11357-025-01589-7 (PMC12181524; doi:10.1007/s11357-025-01589-7)
Supplement: Supplementary file 1 — Supplementary file1 (DOCX 37 KB) [file 11357_2025_1589_MOESM1_ESM.docx]

|  | **Predicted ML-AAC-24 categories** | | | |
| --- | --- | --- | --- | --- |
|  | Low | | Moderate | High |
| Model 3 + hip BMD + prior myocardial infarction or cerebrovascular disease (n=8529) | n=3393 | n=2823 | | n=2313 |
|  | Ref. | 1.33 (1.10-1.60)* | | 1.52 (1.25-1.86)* |
| Model 2 excluding prior fallers (n=7233) | n=2961 | n=2377 | | n=1895 |
|  | Ref. | 1.45 (1.18-1.78)* | | 1.63 (1.31-2.03)* |

**Supplementary Table 1.** Hazard ratio (95% CI) for a fall-associated hospitalisation by severity of predicted abdominal aortic calcification (ML-AAC24).

Model 2 adjusted for age, sex, body mass index, tobacco use, high alcohol intake, income, rural residence, ethnicity; diagnoses of diabetes, hypertension, medication use in the year prior to index date (glucocorticoid, statin, nonselective beta blocker, selective beta blocker, angiotensin receptor blocker, ACE inhibitor, aldosterone blocker, loop diuretic, thiazide diuretic, digoxin, calcium channel blocker, long acting nitrate and oral anticoagulant); and Model 3: Model 2 + falls in the year prior to index date. *Indicates p<0.05 compared to low ML-AAC24.

**Supplementary Table 2.** Hazard ratios (95%CI) for fall-associated hospitalisation by machine learning derived abdominal aortic calcification categories (ML-AAC24) in men and women separately.

|  | Predicted ML-AAC-24 | Fall-associated hospitalizations, n(%) | Model 1 | Model 2 | Model 3 |
| --- | --- | --- | --- | --- | --- |
|  | Low  n=158 | 14 (8.9) | Ref (1.0) | Ref (1.0) | Ref (1.0) |
| Men | Moderate  n=198 | 20 (10.1) | 1.17 (0.59-2.33) | 1.22 (0.58-2.55) | 1.20 (0.57-2.53) |
|  | High  n=156 | 15 (9.6) | 1.13 (0.53-2.41) | 1.30 (0.58-2.92) | 1.19 (0.52-2.73) |
| Women | Low  n=3242 | 191 (5.9) | Ref (1.0) | Ref (1.0) | Ref (1.0) |
|  | Moderate  n=2642 | 252 (9.5) | 1.52 (1.26-1.84)* | 1.41 (1.16-1.71)* | 1.39 (1.14-1.69)* |
|  | High  n=2169 | 258 (11.9) | 1.96 (1.61-2.39)* | 1.67 (1.36-2.05)* | 1.65 (1.34-2.02)* |

Model 1 adjusted for age; Model 2 adjusted for Model 1 + body mass index, tobacco use, high alcohol intake, income, rural residence, ethnicity; diagnoses of diabetes, hypertension, medication use in the year prior to index date (glucocorticoid, statin, nonselective beta blocker, selective beta blocker, angiotensin receptor blocker, ACE inhibitor, aldosterone blocker, loop diuretic, thiazide diuretic, digoxin, calcium channel blocker, long acting nitrate and oral anticoagulant); and Model 3: Model 2 + falls in the year prior to index date. ^*^Indicates p<0.05 compared to low ML-AAC24.

Manitoba de-identified VFA images (2010-2017)
(n=13395)

Excluded (n=653) due to; uncertain vertebral fracture (n=393), pathological fracture (n=5) and poor image quality (n=255)

De-identified VFA images
(n=12742)

Multiple VFA images from the same patient or no linked health outcome data after March 2017

(n=1587)

Unique individuals with VFA-images from their first clinical visit with linked incident falls data (n=11155)

Randomly selected to develop the ML-AAC24 algorithm

(n=2590)^1^

Unique individuals with VFA images not used to develop ML-AAC24 with linked incident falls data
(n=8565)

**Supplementary Figure 1**: Study flow diagram.

^1^ development of the machine learning-derived algorithm for the assessment of abdominal aortic calcification (ML-AAC24) is detailed in reference 17. Vertebral fracture assessment, VFA.
